# Gender and Musculoskeletal Comorbidity Impact on Physical Functioning in Elderly after Hip Fracture: The Role of Rehabilitation

**DOI:** 10.3390/healthcare8010031

**Published:** 2020-02-04

**Authors:** Katarina Radosavljevic, Gordana Dragovic-Lukic, Dejan Nikolic, Pavle Radovic, Biljana Milicic, Natasa Radosavljevic

**Affiliations:** 1 Faculty of Medicine, University of Belgrade, 11000 Belgrade, Serbia; katarina.r12@gmail.com (K.R.); denikol27@gmail.com (D.N.); 2 Department of Pharmacology, Clinical Pharmacology and Toxicology, Faculty of Medicine, University of Belgrade, 11000 Belgrade, Serbia; gordana.dragovic@med.bg.ac.rs; 3 Physical Medicine and Rehabilitation Department, University Children’s Hospital, 11000 Belgrade, Serbia; 4 Institute for Rehabilitation, 11000 Belgrade, Serbia; primpavle@yahoo.com; 5 Faculty of Dental Medicine, University of Belgrade, 11000 Belgrade, Serbia; biljana.milicic@stomf.bg.ac.rs; 6 King Abdulaziz Hospital, 26521 Qurwa, Taif 26521, Saudi Arabia

**Keywords:** gender, musculoskeletal comorbidity, physical functioning, aged adults, rehabilitation

## Abstract

The study aim was to evaluate the effects of presence and level of musculoskeletal impairment along with gender on physical functioning outcome after the rehabilitation program in aged adults with a hip fracture. We analyzed 203 elderly people with hip fractures above 65 years of age that were treated after the hip surgery. According to the time of examination, patients were tested three times: at admission, discharge, and at three months post-discharge. Musculoskeletal impairments were analyzed, and for the estimation of severity of degree impairment, we used a cumulative index rating scale for geriatrics (CIRS-G). Regarding the gender, we separately analyzed males and females. To evaluate physical functioning of aged adults after a hip fracture, we used the physical functioning component (PFC) from the quality of life (SF-36) questionnaire. For males, on all three occasions we found non-significant differences were found in SF-36 PFC values between different degrees of CIRS-G musculoskeletal impairment. A significant difference was noticed in females three months post-discharge. Effects size of different examination periods for every CIRS-G severity degree of musculoskeletal impairment were high, where males had higher values for severity degrees 1 and 2, and females had higher values for severity degrees 0 and 3. Our findings might suggest that there is a certain degree of different rehabilitation treatment effects for males versus females. Moreover, it might be assumed that other factors could influence different degrees of functional improvement and outcome of individuals after a hip fracture with musculoskeletal impairment.

## 1. Introduction

Previously, it was pointed out that around half of patients with a hip fracture will improve physical functioning to pre-fracture levels [1]. Improving mobility, particularly in aged adults after a hip fracture, is of great importance in order to reduce complications. However, despite the fact that there were numerous strategies proposed for mobility improvement, so far there is insufficient evidence for the best strategies that might be implemented for mobility in patients with a hip fracture [2]. The cornerstone of in-hospital rehabilitation for patients admitted and treated for hip fracture is basic mobility enhancement. Numerous studies evaluated predictors that might be associated with mobility and functional improvement during the period of in-hospital rehabilitation [3,4,5,6]. Further, it was noticed that gender might be a factor that could influence rehabilitation outcome in aged adults after a hip fracture, thus affecting therapeutic decision making and requiring adjustments for certain interventions regarding better functional restoration [7,8].

Therefore, the aim of our study was to evaluate the effects of presence and level of musculoskeletal impairment along with gender on physical functioning outcome after a rehabilitation program in aged adults with a hip fracture.

## 2. Materials and Methods

### 2.1. Study Group

In a longitudinal study, we analyzed 203 elderly patients above 65 years of age (54 males and 149 females), that were treated after hip surgery for hip fracture. Inclusion criteria were: individuals age 65 or above with first time hip fracture due to a fall, who underwent a surgery and were admitted to the in-patient rehabilitation. Prior to inclusion in the study, patients were informed about the study protocol and informed consent was obtained. The study followed the principles of good clinical practice and conformed to the declaration of Helsinki. The study was approved by institutional review board of faculty of medicine in Belgrade (440/IV-6).

Regarding the time of examination, eligible participants that were included into the study were tested on three occasions: at admission, at discharge, and at three months post-discharge. Musculoskeletal impairments were analyzed, and for the estimation of severity degree impairment we used cumulative index rating scale for geriatrics (CIRS-G) in the range between 0–4 only at admission, where 0- refers to the condition with no impairment, 1- refers to mild, 2- refers to moderate, 3- refers to severe, and 4- refers to extremely severe impairment [5,9]. Regarding gender, we separately analyzed males and females.

Prescription of a rehabilitation program for eligible participants was individually assessed, taking into consideration the patient’s motor functional status. The program was conducted five times per week by a licensed physiotherapist under the supervision of a board-certified specialist of physical medicine and rehabilitation.

To evaluate physical functioning of studied patients after hip fracture, we used the physical functioning component (PFC) from the quality of life (SF-36) questionnaire [10].

### 2.2. Statistical Analysis

Frequencies of patients for different degrees of CIRS-G for musculoskeletal impairment in total and regarding gender were presented as whole numbers (N) and percentages (%). Mean values and median (MV and *Med*) with standard deviation and inter quartile range (SD and *IQR*) were used to present values of SF-36 PFC for different degrees of CIRS-G for musculoskeletal impairment in different times of observation. Chi squared test was used to assess statistical significance of frequency distributions between genders. Numeric data (SF-36 PFC) were tested for normal distribution using the Kolmogorov–Smirnov test. All our data were nonparametric. For evaluation of SF-36 PFC values among more than two different times of observation the Friedman test was used, while the Wilcoxon test was performed for evaluation between two observational times. For estimation of SF-36 PFC values between patients with different CIRS-G severity degrees, in observed periods of time the Kruskal–Wallis test was used, while between groups the Mann–Whitney test was used. 

For an explanation of relative variability, we used coefficient of variation (V) that was expressed as percent and calculated as the ratio between standard deviation and mean value for the observed parameter. For evaluation and quantification of variability that can be explained between different CIRS-G severity degrees and the values of physical functioning of SF-36 questionnaire for evaluated CIRS-G parameters (musculoskeletal impairment), we introduced η2 = sum of squares (between groups)/sum of squares (total) × 100, where sum of squares were gained from the one-way ANOVA test and results were presented as a percentage (%) [5]. For evaluation and quantification of variability that can be explained between different times of evaluation for the same level of CIRS-G severity musculoskeletal impairment and the values of physical functioning of SF-36 questionnaire, we introduced η2 as well. 

A *p* value < 0.05 was required to reject the null hypothesis. Statistical analyses were performed using SPSS software version 26.0 (Chicago, IL, USA).

## 3. Results

There is no statistical significance in frequencies of participants regarding gender with different degrees of CIRS-G (Table 1). In all CIRS-G subgroups, females were frequently present. 

**Figure 1:**
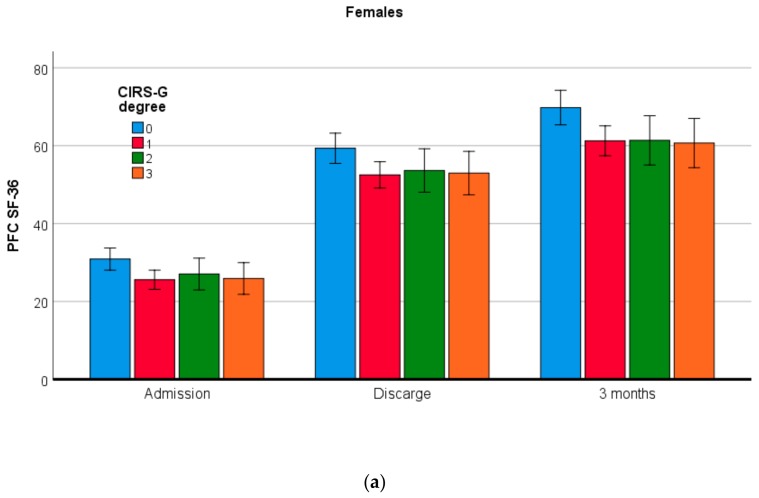

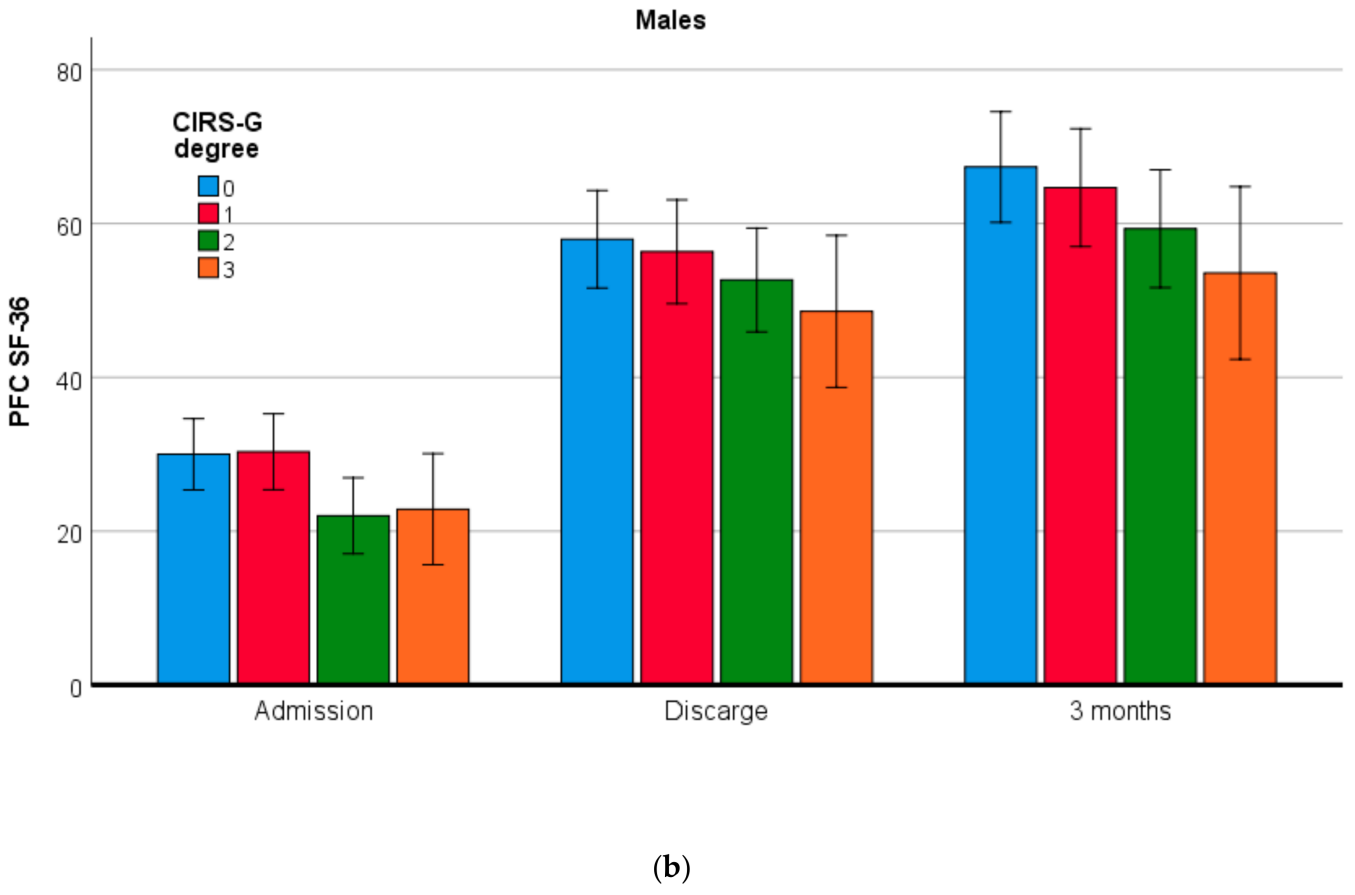
Mean values with 95% CI of physical functioning component (PFC) SF-36 for different CIRS-G degrees on three occasions in: (**a**) females; (**b**) males.

There is a significant difference in SF-36 PFC values between different degrees of CIRS-G musculoskeletal impairment on all 3 examination periods (Admission, *p* = 0.018; Discharge, *p* = 0.028; three months, *p* = 0.004) (Table 2). Performing between groups analysis, there is statistical significance between participants with CIRS-G severity degree 0 and those with severity degrees 1, 2, and 3 in all three times of examination. For males, on all three examination occasions we found a non-significant difference in SF-36 PFC values between different degrees of CIRS-G musculoskeletal impairment (Admission, *p* = 0.053; Discharge, *p* = 0.286; three months, *p* = 0.181), while for females a significant difference was noticed only three months after discharge (*p* = 0.022) (Figure 1, Table 2). In analysis of obtained differences in SF-36 PFC, for females, three months after discharge, significantly higher values of this parameter were noticed in those with CIRS-G severity degree 0 versus females with higher severity degrees (1, 2, and 3).

Regarding relative variability, except for CIRS-G degree 2, there was a decrease among different times of observation (Admission, three months), while effect sizes of CIRS-G severity degree for musculoskeletal impairment were low (Table 3). Relative variability values differed between genders, and effect sizes of CIRS-G severity degree for musculoskeletal impairment were higher in males on all three examination periods, particularly for the admission examination period (η^2^_Males_ = 14.22% and η^2^_Females_ = 5.61%) (Table 3). Effect sizes of different examination periods for every CIRS-G severity degree of musculoskeletal impairment were high, where males had higher values for severity degrees 1 (η^2^_Males_ = 67.10% and η^2^_Females_ = 55.38%) and 2 (η^2^_Males_ = 63.19% and η^2^_Females_ = 59.39%), and females for severity degree 0 (η^2^_Males_ = 60.26% and η^2^_Females_ = 65.29%) and 3 (η^2^_Males_ = 53.05% and η^2^_Females_= 57.64%) (Table 3).

For the entire study population and females, there was a significant difference between different times of observation in patients with the same CIRS-G severity degree (*p* = 0.000) (Table 4). Males had significant differences in physical components of SF-36 between admission and at discharge for all CIRS-G severity degrees, while a non-significant difference was found in males for CIRS-G severity degree 3, between discharge and three months post-discharge (*p* = 0.083) (Table 4).

## 4. Discussion

The functioning of patients who suffered a hip fracture is affected multidimensionally, particularly in the physical, social, and emotional domains [11]. It was previously reported that there are outcome differences between genders after hip fracture, stating that males tend to have higher mortality rates and are more likely to require assistive devices for mobilization [12]. Further, in the study of Woodward et al., it was demonstrated that gender in patient groups who underwent surgical intervention after hip fracture had an impact on coordinated stability [12].

We have demonstrated that physical functioning in females significantly differed with regards to the presence and the level of CIRS-G severity degree three months post-discharge, while males presented with no significant differences in physical functioning.

Considering relative variability of SF-36 PFC values across different degrees of CIRS-G severity degree, in our study we demonstrated lower variability in those patients on all occasions of evaluation that were without musculoskeletal impairment, while those with different severity degrees had a higher relative variability, where the highest CIRS-G severity degree had the highest relative variability at discharge and three months post-discharge. However, when such parameters were analyzed with regards to gender, the pattern did not follow such observation for males and all three evaluation times. Contrary to the entire study population, a lower relative variability was noticed for those males that had CIRS-G severity degree 1. Females followed the pattern of lower relative variability in comparison to those that were without and those that had musculoskeletal impairment, where the highest CIRS-G severity degree had the highest relative variability only at discharge. These findings might suggest that there is a certain degree of different rehabilitation treatment effects for males versus females. Moreover, it might be assumed that other factors could influence different degrees of functional improvement and outcome of individuals after a hip fraction with musculoskeletal impairment. Previously, it was stated that males with cognitive dysfunction had an increased risk for loss of walking ability versus females [13]. In the study of Mizrahi et al. [14] it was noticed that females are an independent predictor for higher functional independence measure score on discharge versus males. Our findings could point to the assumption that males with lower CIRS-G severity degree (particularly severity degree 1), and females without musculoskeletal impairments have more homogenous response in terms of physical functioning. 

The effects sizes of CIRS-G severity degrees for musculoskeletal impairment on SF-36 PFC values were low in all of the evaluated groups. However, the effects sizes regarding different times of observation for different CIRS-G severity degrees from musculoskeletal impairment on SF-36 PFC values were high. This might better explain the effects of rehabilitation treatment on physical functioning than the degree of musculoskeletal impairment. The same applies when effect sizes were compared separately for males and females in our study. However, it should be noted that there is a decrease by more than half in effect sizes of CIRS-G severity degrees for musculoskeletal impairment on SF-36 PFC between admission and on discharge from the rehabilitation program for males. The present differences in such parameters between genders could assume that gender might be a potential factor in rehabilitation outcome response to improvement in physical functioning for patients with musculoskeletal impairment.

Furthermore, we have demonstrated that effect sizes of physical functioning values at different examination periods were higher (above 50%) for every CIRS-G severity degree, where males had higher values than females. Moreover, for every CIRS-G severity degree, there is a difference in the degree of the correlation between physical functioning changes between males and females, particularly in the period between discharge and at follow-up. This assumes that there could be different responses to the rehabilitation treatment between genders after discharge from the rehabilitation unit, stressing the necessity for an individual approach in the planning of rehabilitation treatment of these patients. 

## 5. Conclusions

Given the facts above, we believe that better understanding of certain predictors and confounding factors of functional outcome in aged adults after hip fracture could result in adopting better treatment strategies, particularly rehabilitation programs, and will help practitioners in clinical practice to apply the best possible clinical decision-making strategies. This will ultimately reduce overall mortality and improve the quality of life of aged adults after a hip fracture.

## Figures and Tables

**Table 1:** Frequencies of comorbidities.

CIRS-G (Degree)	Musculoskeletal Impairment			
	Total N (%)	Males N (%)	Females N (%)	Males/Females *p* Value *
0	62	17 (27.4)	45 (72.6)	0.141
1	75	15 (20.0)	60 (80.0)	
2	37	15 (40.5)	22 (59.5)	
3	29	7 (24.1)	22 (75.9)	
4	0	0 (0)	0 (0)	
∑	203	54 (26.6)	149 (73.4)	-

* Chi squared test.

**Table 2:** SF-36 PFC mean values in different time of observation for separate CIRS-G parameters regarding severity degree.

Study Participants	CIRS-G (Degree)	Period of Observation			*p* Value *
		Group 1 MV ± SD *(Med (IQR))*	Group 2 MV ± SD *(Med (IQR))*	Group 3 MV± SD *(Med (IQR))*	
**Overall**	0	30.65 ± 9.90 (*35* (*10*))	58.95 ± 13.00 (*65* (*15*))	69.11 ± 13.84 (*75* (*15*))	0.000
	1	26.53 ± 9.69 (*25* (*15*))	53.27 ± 13.62 (*55* (*20*))	61.93 ± 15.62 (*65* (*25*))	0.000
	2	25.00 ± 10.14 (*25* (*15*))	53.24 ± 11.80 (*55* (*20*))	60.54 ± 15.13 (*65* (*18*))	0.000
	3	25.17 ± 9.01 (*25* (*13*))	51.90 ± 14.04 (*55* (*15*))	58.97 ± 15.55 (*60* (*18*))	0.000
*p* value **		0.018	0.028	0.004	
**Male**	0	30.00 ± 10.61 (*30* (*13*))	57.94 ± 13.93 (*60* (*15*))	67.35 ± 14.91 (*75* (*18*))	0.000
	1	30.33 ± 8.34 (*30* (*5*))	56.33 ± 10.08 (*55* (*20*))	64.67 ± 12.88 (*65* (*20*))	0.000
	2	22.00 ± 11.15 (*20* (*15*))	52.67 ± 11.32 (*50* (*20*))	59.33 ± 15.57 (*65* (*20*))	0.000
	3	22.86 ± 8.09 (*25* (*10*))	48.57±12.49 (*55* (*20*))	53.57 ± 18.42 (*65* (*35*))	0.000
***p* value**		0.053	0. 286	0.181	
**Female**	0	30.89 ± 9.73 (*35* (*10*))	59.33 ± 12.77 (*65* (*15*))	69.78 ± 13.52 (*75* (*18*))	0.000
	1	25.58 ± 9.83 (*25* (*20*))	52.50 ± 14.34 (*55* (*24*))	61.25 ± 16.25 (*65* (*25*))	0.000
	2	27.05 ± 9.08 (*30* (*15*))	53.64 ± 12.36 (*55* (*20*))	61.36 ± 15.13 (*65* (*20*))	0.000
	3	25.91 ± 9.34 (*25* (*15*))	52.95 ± 14.61 (*55* (*20*))	60.68 ± 14.58 (*60* (*21*))	0.000
***p* value**		0.058	0.084	0.022	

MV: mean value; SD: standard deviation; Med: median; IQR: inter quartile range; * Friedman test; ** Kruskal Wallis test.

**Table 3:** Relative variability and effect sizes of evaluated parameters in study sample.

Study Participants	CIRS-G (Degree)	Variability (V-%)			η^2^ (%)
		Admission	Discharge	3 months	
**Overall**	0	32.30	22.05	20.02	63.78
	1	36.52	25.57	25.22	66.97
	2	40.56	22.16	24.99	60.59
	3	35.80	27.05	26.37	55.87
**η^2^(%)**		5.35	4.38	6.39	
**Male**	0	35.37	24.04	22.14	60.26
	1	27.50	17.89	19.92	67.10
	2	50.68	21.50	26.24	63.19
	3	35.39	25.72	34.38	53.05
**η^2^(%)**		14.22	7.00	9.44	
**Female**	0	31.50	21.52	19.38	65.29
	1	38.43	27.31	26.53	55.38
	2	33.57	23.04	24.66	59.39
	3	36.05	27.59	24.03	57.64
**η^2^(%)**		5.61	4.75	6.65	

**Table 4:** Correlations of SF-36 PFC values in defined severity degree of CIRS-G regarding the time of observation.

Study Participants	CIRS-G (Degree)	Time Groups	Admission *p* Value *	Discharge *p* Value *
Overall	0	Discharge	0.000	
		3 months	0.000	0.000
	1	Discharge	0.000	
		3 months	0.000	0.000
	2	Discharge	0.000	
		3 months	0.000	0.000
	3	Discharge	0.000	
		3 months	0.000	0.000
Male	0	Discharge	0.000	
		3 months	0.000	0.000
	1	Discharge	0.001	
		3 months	0.001	0.001
	2	Discharge	0.001	
		3 months	0.001	0.010
	3	Discharge	0.018	
		3 months	0.018	0.083
Female	0	Discharge	0.000	
		3 months	0.000	0.000
	1	Discharge	0.000	
		3 months	0.000	0.000
	2	Discharge	0.000	
		3 months	0.000	0.000
	3	Discharge	0.000	
		3 months	0.000	0.000

* Willcoxon test.
